# On the Validation of a Multiple-Network Poroelastic Model Using Arterial Spin Labeling MRI Data

**DOI:** 10.3389/fncom.2019.00060

**Published:** 2019-09-03

**Authors:** Liwei Guo, Zeyan Li, Jinhao Lyu, Yuqian Mei, John C. Vardakis, Duanduan Chen, Cong Han, Xin Lou, Yiannis Ventikos

**Affiliations:** ^1^Department of Mechanical Engineering, University College London, London, United Kingdom; ^2^School of Life Science, Beijing Institute of Technology, Beijing, China; ^3^Department of Radiology, Chinese PLA General Hospital, Beijing, China; ^4^Department of Computer Science, INSIGNEO Institute, University of Sheffield, Sheffield, United Kingdom; ^5^Department of Neurosurgery, The Fifth Medical Centre of PLA General Hospital, Beijing, China

**Keywords:** poroelasticity, multiple fluid networks, finite element method, cerebral blood flow, blood perfusion, arterial spin labeling, magnetic resonance imaging, brain

## Abstract

The Multiple-Network Poroelastic Theory (MPET) is a numerical model to characterize the transport of multiple fluid networks in the brain, which overcomes the problem of conducting separate analyses on individual fluid compartments and losing the interactions between tissue and fluids, in addition to the interaction between the different fluids themselves. In this paper, the blood perfusion results from MPET modeling are partially validated using cerebral blood flow (CBF) data obtained from arterial spin labeling (ASL) magnetic resonance imaging (MRI), which uses arterial blood water as an endogenous tracer to measure CBF. Two subjects—one healthy control and one patient with unilateral middle cerebral artery (MCA) stenosis are included in the validation test. The comparison shows several similarities between CBF data from ASL and blood perfusion results from MPET modeling, such as higher blood perfusion in the gray matter than in the white matter, higher perfusion in the periventricular region for both the healthy control and the patient, and asymmetric distribution of blood perfusion for the patient. Although the partial validation is mainly conducted in a qualitative way, it is one important step toward the full validation of the MPET model, which has the potential to be used as a testing bed for hypotheses and new theories in neuroscience research.

## Introduction

**Figure 1:**
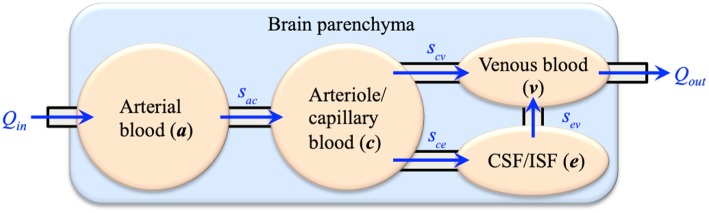
The four-network poroelastic model (4-MPET) used for mechanistic modeling of the brain.

Computational modeling has shown great potential in biomedical engineering research. The main advantage is that computational methods can translate mathematical formulations that describe the inherent complexity of biological systems into computer programs and solve them in a timely manner. Many software suites have been developed for mechanistic modeling of biological systems, such as SfePy (Rohan and Cimrman, 2012), FEBio (Maas et al., 2012), and FEniCS (Logg et al., 2012). In this respect, one of the promising tools is applying the multiple-porosity/multiple-permeability poroelastic model for modeling of fluid transport and tissue deformation in the brain, which is called the Multiple-network PoroElastic Theory (MPET). The brain parenchyma is treated as a deformable solid matrix, permeated by multiple fluid networks (Tully and Ventikos, 2011). In general, the number of fluid networks can be customized to specific research. For current brain modeling, four coupled fluid networks are taken into account: an arterial network (*a*), an arteriole/capillary network (*c*), a cerebrospinal fluid/interstitial fluid (CSF/ISF) network (*e*) and a venous network (*v*). The directional flows between the fluid networks are shown in Figure 1, which link all four fluid compartments together to form a coupled and integrated fluid domain. The separation of arterial and arteriole networks is based on the consideration of different resistances between large and small arteries. Similar implementation was adopted in the modeling of coronary blood flow in the heart (Smith et al., 2002; Lee and Smith, 2012), where the arterial tree consists of several compartments. In general, arterioles are defined as the primary resistance vessels that enter an organ to distribute arterial blood into capillary beds, which provides more than 80% of the resistance to blood flow in the body (Mulvany and Aalkjaer, 1990; Christensen and Mulvany, 2001; Martinez-Lemus, 2011). Therefore, the arterial blood compartment is further segmented into a high-pressure arterial network and a lower-pressure arteriole/capillary network (Tully and Ventikos, 2011).

The MPET theory has been successfully used in the modeling of biomechanical problems, e.g., hydrocephalus (Levine, 2008; Tully and Ventikos, 2011; Sobey et al., 2012), cerebral oedema (Vardakis et al., 2016), and Alzheimer's disease (Guo et al., 2018; Vardakis et al., 2019). However, there still lacks thorough and rigorous validation using experimental and clinical data. Computational tools developed in other fields of biomedical engineering has shown that once sufficiently validated, they can be used as testing beds for clinical research, e.g., analyzing risks and exploring new treatments for diseases (Chen et al., 2018).

The MPET model can generate a wide range of output results, such as the pressure and Darcy velocity (filtration velocity) of fluids and brain tissue deformation. This gives the users great advantage to have a full picture to understand the biomechanical mechanisms at multiple scales. However, it also brings difficulty to the validation of the model. Due to the complexity of the algorithms and the large number of parameters needed to define the MPET model, it is not feasible to validate the entire model in one validation test. Therefore, a series of validation tests need to be designed and conducted to fully validate the numerical model and this paper aims to be as one step in this process.

One important output that can be seen from MPET modeling is blood perfusion, which is represented by the filtration velocity of the arteriole/capillary blood compartment. Blood perfusion in the brain can be quantified by cerebral blood flow (CBF), which is an important parameter to define brain function. For example, by quantifying regional CBF, Chen et al. (2011) demonstrated that normal aging has different effects on regional CBF and gray matter atrophy, although age-related reductions are more common in cortical perfusion than subcortical CBF. Lassila et al. (2018) observed evidence of hypoperfusion being associated with mild cognitive impairment (MCI) status. Moreover, much research have been conducted to explore the possibility of using CBF as a biomarker for early diagnosis of Alzheimer's disease (AD) and other dementias. One of the findings is decreased blood flow in praecuneus and/or posterior cingulum, and in the lateral parietal cortex (Alsop et al., 2010); other studies in AD (Alsop et al., 2008; Dai et al., 2009; Fleisher et al., 2009) found elevated CBF in the hippocampus. The hippocampus is associated with spatial and episodic memory; for example, reduced hippocampal volume results in an amnestic syndrome, which is a core feature of AD (Halliday, 2017).

Several methods can be used to measure CBF, such as computed tomography perfusion (CT perfusion), positron emission tomography (PET), and single-photon emission computed tomography; however, CBF measured by different methods normally cannot be compared directly (Kudo et al., 2003; Guibert et al., 2013). In addition to the methods mentioned above, an increasingly popular method to quantify CBF is to use arterial spin labeling (ASL) magnetic resonance imaging (MRI). Arterial spin labeling (ASL) is a non-invasive imaging technique using standard magnetic resonance imaging (MRI) equipment. The basic idea is that an MRI image can be sensitized to the effect of inflowing blood spins, if the spins are in a different magnetic state from that of the static tissue. The ASL technique based on this idea uses magnetically labeled arterial blood water as a nominally diffusible tracer for blood flow measurements. There are several schemes for labeling arterial blood water, including continuous labeling, pseudo continuous labeling, and pulsed labeling (Calamante et al., 1999). Continuous ASL means continuously rotating arterial spins as they pass a labeling plane just beneath the imaged region (Williams et al., 1992). Pulsed labeling means rotating arterial spins in a slab of tissue at one time (Wong et al., 1997), which is most often used in functional magnetic resonance imaging (fMRI). The physiological basis for the MRI contrast mechanisms of ASL is well-known so it provides a biomarker for brain function that is portable across scanning platforms or time (Alsop et al., 2010). ASL perfusion MRI has been used as a diagnostic tool in clinical practice (Detre et al., 2012; Alsop et al., 2015), and also in human neuroscience research (Detre et al., 2009; Shin et al., 2013).

The objective of this paper is to partially validate the blood perfusion obtained from 4-MPET modeling using CBF data from ASL images. The paper is organized in the following way. First, the image collection, including T1-weighted MRI and ASL MRI, is introduced. The T1-weighted MRI is segmented to create geometry and mesh for numerical modeling. Second, the numerical formulation of the 4-MPET model, and the boundary conditions and parameters used for modeling are described. Third, the numerical results of blood perfusion are compared with CBF data obtained from ASL images, and similarities and differences are discussed. Lastly, some conclusions are drawn from the validation tests and future work is suggested.

## Materials and Methods

### Clinical Data Collection and Processing

The clinical data were collected at the People's Liberation Army (PLA) General Hospital in Beijing, China. Two subjects—one healthy control and one patient with unilateral middle cerebral artery (MCA) stenosis, are included in this paper. The ethics committee of the PLA General Hospital approved the study and both participants gave informed consent prior to participation in the study. After data collection, T1-weighted (T1w) MR images were segmented to create three-dimensional geometries and meshes of parenchymal tissue and the cerebral ventricles for numerical modeling; Arterial spin labeling (ASL) MR images were processed to generate cerebral blood flow (CBF) maps, which were used for validation of the numerical results.

#### T1-Weighted MRI

**Figure 2:**
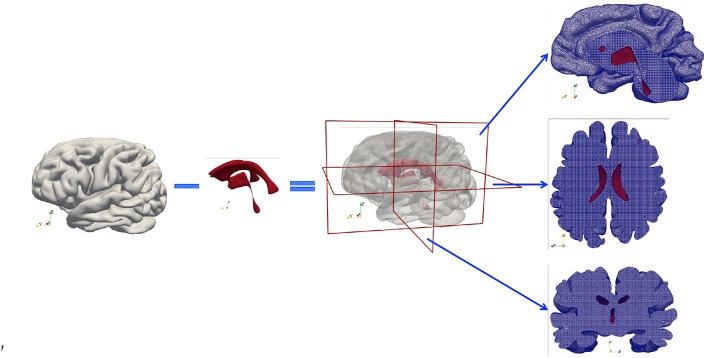
Subject-specific brain geometry obtained by segmentation of T1w MR images and the application of the Boolean operation. The finished model is a volumetric domain with a cavity representing the ventricles. The tetrahedral mesh created for numerical modeling is demonstrated by cross-sections cut in three orthogonal directions. The red color represents the ventricular wall.

**Figure 3:**
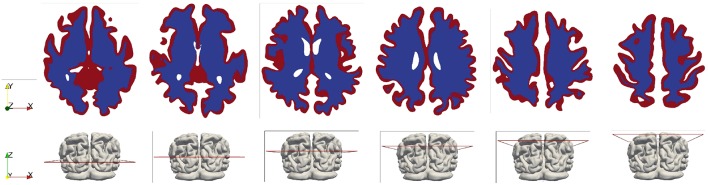
White and gray matter in the brain model (shown in the horizontal cross-sectional slices). The white matter is represented by the blue color and the gray matter is represented by the red color.

A high-resolution T1w dataset using a 3D Ax FSPGR (fast spoiled gradient-recalled echo) sequence was acquired and used to generate masks. The scan parameters were as follows: repetition time/echo time (TR/TE), 5.9960/2.5400 ms; inversion time, 450 ms; bandwidth, ±16 kHz; slice thickness, 1 mm; matrix, 512 × 512; flip angle, 15°. Subsequently, these MR images were segmented to create anatomically accurate three-dimensional brain geometries using FreeSurfer (Fischl, 2012). The emphasis here is to capture detailed cortical and subcortical features, such as the gray and white matter and the cerebroventricular system. Initially two closed surfaces were created from segmentation—the outer surface represents the cortical surface of the brain parenchyma and the inner surface represents the ventricular wall. Next, the volume formed by the ventricular wall was deducted from the volume formed by the cortical surface via a Boolean operation, so the final volumetric domain used for numerical modeling is the brain parenchyma between the cortical surface and the ventricular wall (Figure 2). Furthermore, the brain parenchyma was segmented into separate regions of white matter and gray matter to characterize their different mechanical properties (Figure 3), which makes this model more realistic than previous models using homogeneous representations of the brain parenchyma (Guo et al., 2018). The final geometric model was discretized into 4-node tetrahedra elements using ANSYS (ANSYS, Inc., Canonsburg, USA). The mesh size satisfies the criterion proposed from mesh sensitivity tests in a previous paper (Guo et al., 2018) to make sure the numerical results are convergent.

#### Arterial Spin Labeling MRI

In order to obtain cerebral blood flow (CBF) data for the validation of numerical results, the participants were scanned using 3D pseudo-continuous arterial spin labeling (pCASL) technology (Discovery 750, GE Healthcare). The technical parameters are listed as follows: sequence repetition time/echo time (TR/TE), 5,327/10.5 ms; field of view, 240 × 240 mm; matrix size, 128 × 128; number of slices, 36; slice thickness, 4 mm; labeling duration, 1,500 ms; post-labeling delay, 1,525 ms; and number of excitation, 2; background suppressed. Then the ASL perfusion maps were expressed as cerebral blood flow (CBF) by the supporting software of the MR scanner. A skull stripping function was implemented in the ASL image processing workflow so the scalp tissues can be removed on the CBF maps by creating a tissue mask from T1-weighted images (Deibler et al., 2008). The CBF results are compared with numerical results in section Results and Discussion.

### Multiple-Network Poroelastic Model

#### Finite Element Model

The multiple-network poroelastic model incorporates mechanical equilibrium for elastic deformation, mass conservation of fluids and Darcy's law for fluid flow in a coupled manner. The governing equations of the 4-MPET model are listed as follows, where the primitive variables are the displacement of the parenchymal tissue (**u**) and the pressures of the four fluid networks *p*_*i*_ (*i* = *a, c, e, v*).

(1) $$\begin{array}{l} G\nabla^{2}\text{u}+\left(G+\lambda \right)\nabla \varepsilon =\alpha_{a}{\nabla p}_{a}+\alpha_{c}{\nabla p}_{c}+\alpha_{e}{\nabla p}_{e}+\alpha_{v}{\nabla p}_{v} \end{array}$$

(2) $$\begin{array}{l} S_{a}\frac{\partial p_{a}}{\partial t}+\alpha_{a}\frac{\partial \varepsilon }{\partial t}=\frac{k_{a}}{\mu_{a}}\nabla^{2}p_{a}+s_{ca} \end{array}$$

(3) $$\begin{array}{l} S_{c}\frac{\partial p_{c}}{\partial t}+\alpha_{c}\frac{\partial \varepsilon }{\partial t}=\frac{k_{c}}{\mu_{c}}\nabla^{2}p_{c}+\left(s_{ac}+s_{ec}+s_{vc}\right) \end{array}$$

(4) $$\begin{array}{l} S_{e}\frac{\partial p_{e}}{\partial t}+\alpha_{e}\frac{\partial \varepsilon }{\partial t}=\frac{k_{e}}{\mu_{e}}\nabla^{2}p_{e}+\left(s_{ce}+s_{ve}\right) \end{array}$$

(5) $$\begin{array}{l} S_{v}\frac{\partial p_{v}}{\partial t}+\alpha_{v}\frac{\partial \varepsilon }{\partial t}=\frac{k_{v}}{\mu_{v}}\nabla^{2}p_{v}+\left(s_{cv}+s_{ev}\right) \end{array}$$

Equation 1 is the equilibrium equation, which describes the momentum balance in the porous medium. Here, **u** is the displacement of the tissue; *p*_*i*_ is the pressure in each fluid network; *G* is the shear modulus; λ is the Lamé's constant; ε is the dilatational strain; α_*i*_ is the Biot–Willis coefficient for each fluid network which satisfies ϕ ≤ α_*a*_ + α_*c*_ + α_*e*_ + α_*v*_ ≤ 1 (Berryman, 1992; Wang, 2000), where ϕ is the total porosity. In this paper, only four fluid networks are considered so the total porosity ϕ equals the sum of the porosities of the four individual networks (Bai et al., 1993; Tully and Ventikos, 2011). It is worth noting that the shear modulus *G* and the Lamé's constant λ are not constant in the domain; they have different values in the gray matter and the white matter. Body forces (e.g., gravity) and inertia terms are neglected in the governing equations based on the assumption that the acceleration frequencies are low in biological flows (Tully and Ventikos, 2011; Chou et al., 2016). It should also be noted that the cross-porosity storage effect (Mehrabian and Abousleiman, 2014) is not considered in this paper due to the lack of experimental data to quantify the parameters in a physiologically realistic way (Vardakis et al., 2017).

Equations 2–5 are continuity equations, which describe the mass balance of the four fluid networks, respectively. *S*_*i*_ is the specific storage; *k*_*i*_ is the permeability for each of the four fluid networks; μ_*i*_ is the viscosity of each fluid. The assumption adopted in this paper is that the four fluid domains are isotropic; therefore *k*_*i*_ is a constant. If spatially varying parameters are available, such as permeability tensors extracted from diffusion-weighted imaging (DWI), the permeability *k* can be defined on a heterogeneous and anisotropic basis (Guo et al., 2018).

The *s*_*ij*_ terms on the right-hand side of Equations 2–5 (also demonstrated in Figure 1) define spatially varying source (*s*_*ij*_ > 0) or sink (*s*_*ij*_ < 0) terms (Tully and Ventikos, 2011; Vardakis et al., 2013), which are assumed to be driven by a hydrostatic pressure gradient of the form, *s*_*ij*_ = ω_*ij*_(*p*_*i*_ – *p*_*j*_), where ω_*ij*_ is the transfer coefficient scaling the flow from network *i* to network *j*. The transfer of fluid between the four fluid networks is derived from physiological considerations (Tully and Ventikos, 2011) and required to obey the law of continuity for the entire domain; hence, directionality between fluid compartments must be accurately specified. These are listed as follows:

1. Directional fluid transport always occurs from the arterial network to the arteriole/capillary network:
  (6) $$\begin{array}{l} s_{ac}=-s_{ca}=|s_{ac}|\geq 0 \end{array}$$
2. Fluid transport from the arteriole/capillary network enter the CSF/ISF network or the venous network:
  (7) $$\begin{array}{l} s_{ce}=-s_{ec}=|s_{ec}|\geq 0 \end{array}$$
  (8) $$\begin{array}{l} s_{cv}=-s_{vc}=|s_{vc}|\geq 0 \end{array}$$
3. CSF flows into the venous compartment:
  (9) $$\begin{array}{l} s_{ev}=-s_{ve}=|s_{ev}|\geq 0 \end{array}$$

Next, the governing equations are discretized by the finite element method and implemented in an in-house Fortran code. Both the displacement field **u** and the pressures of the four fluid networks *p*_*i*_ (*i* = *a, c, e, v*) are approximated in the continuous piecewise linear polynomial space. The discretized form of the equilibrium equation is derived from the principle of minimum potential energy,

(10) $$\begin{array}{l} \text{K}\text{u}-\left({\text{Q}}_{a}{\text{p}}_{a}+{\text{Q}}_{c}{\text{p}}_{c}+{\text{Q}}_{e}{\text{p}}_{e}+{\text{Q}}_{v}{\text{p}}_{v}\right)=\text{F} \end{array}$$

where

(11) $$\begin{array}{l} \text{K}=\int_{\Omega }{\text{B}}^{T}\text{D}\text{B}\text{d}\Omega \end{array}$$

(12) $$\begin{array}{l} {\text{Q}}_{i}=\int_{\Omega }\alpha_{i}{\text{B}}^{T}\text{h}\text{d}\Omega \end{array}$$

(13) $$\begin{array}{l} \text{F}=\int_{\Omega }{\text{N}}^{T}\text{b}\text{d}\Omega +\int_{\Gamma_{N}}{\text{N}}^{T}{\text{t}}_{N}\text{d}\Gamma \end{array}$$

**K** is the stiffness matrix; **Q**_*i*_ is the load on the solid phase contributed from the *i*^th^ fluid network (*i* = *a, c, e, v*); **b** is the vector of body force, which is neglected in this paper; **N** is the matrix of continuous piecewise linear polynomial functions (shape functions); and **t**_*N*_ is the external force acting on the boundary Γ_*N*_.

The continuity equations of the fluid networks are discretized using the method of weighted residuals and the continuous Galerkin formulation. The discretized form of the continuity equation for one of the four fluid networks is,

(14) $$\begin{array}{l} \text{A}\overset{\cdot }{\text{p}}+\text{C}\text{p}=\text{P} \end{array}$$

The elements in matrices **A**, **C**, and vector **P** are

(15) $$\begin{array}{l} A_{ij}=S\int_{\Omega }N_{i}N_{j}\text{d}\Omega \end{array}$$

(16) $$\begin{array}{l} C_{ij}=\frac{k}{\mu }\int_{\Omega }\left(\frac{\partial N_{i}}{\partial x}\frac{\partial N_{j}}{\partial x}+\frac{\partial N_{i}}{\partial y}\frac{\partial N_{j}}{\partial y}+\frac{\partial N_{i}}{\partial z}\frac{\partial N_{j}}{\partial z}\right)\text{d}\Omega \end{array}$$

(17) $$\begin{array}{l} P_{i}=\int_{\Omega }sN_{i}\text{d}\Omega -\alpha \int_{\Omega }\overset{\cdot }{\varepsilon }N_{i}\text{d}\Omega +\int_{\Gamma_{2}}qN_{i}\text{d}\Gamma \end{array}$$

*N*_*i*_ is the continuous piecewise linear polynomial function at node *i*; and *q* is the flux prescribed in the Neumann boundary condition acting on the boundary *Γ*_2_.

The temporal discretization of the governing equations is implemented using the method of weighted residuals. In this paper, an implicit backward Euler scheme is used for time discretization. The final system of discretized governing equations is solved by the standard KSP linear equation solver in the PETSc library (Balay et al., 2018a,b). The highly coupled equations are solved sequentially in a tightly coupled manner, i.e., the pressure and displacement solutions are solved sequentially during a time-step until a convergence tolerance is reached. At the end of each time-step, Darcy's law is used to calculate Darcy velocities (filtration velocities) of the four fluid networks.

(18) $$\begin{array}{l} \text{v}=-\frac{k}{\mu }\nabla p \end{array}$$

where **v** is the Darcy velocity for each of the four fluid compartments, i.e., the volume of fluid crossing a unit area per unit time. It should be noted that the focus of this paper is to validate one of the outputs from 4-MPET modeling—blood perfusion; here the blood perfusion is represented by the Darcy velocity of the arteriole/capillary compartment.

#### Boundary Conditions and Poroelastic Parameters

As illustrated in Figure 2, the simulation domain of the parenchymal tissue is bounded by two surfaces—the outer boundary represents the cortical surface and the inner boundary represents the ventricular wall, both of which need boundary conditions for the solid phase and the four fluid networks, respectively; therefore, a total of 10 boundary conditions are listed in Table 1.

**Table 1:** Boundary conditions used in the 4-MPET modeling.

	**Cortical surface**		**Ventricular wall**	
Displacement	**u** = 0	(19)	No displacement constraints	
Arterial blood	∇*p*_*a*_**n** = *Q*_*a*_	(20)	∇*p*_*a*_**n** = 0	(21)
Arteriole/capillary blood	∇*p*_*c*_**n** = 0	(22)	κ_*c*→*vent*_∇*p*_*c*_**n** = −*Q*_*p*_	(23)
CSF/ISF	*p*_*e*_ = *p*_*v*_ + μ_*e*_*RQ*_0_	(24)	$Q_{p}=\frac{\pi d^{4}}{128\mu_{e}L}\left(p_{e}-p_{e}^{cortial\;surface}\right)-\frac{{4\pi k}_{e}}{\mu_{e}}{\left(r_{1}+u_{1}^{n}\right)}^{2}\nabla p_{e}\text{n}+4\pi {\left(r_{1}+u_{1}^{n}\right)}^{2}\overset{\cdot }{u}$	(25)
Venous blood	*p*_*v*_ = *p*_*bp*_	(26)	∇*p*_*v*_**n** = 0	(27)

**Figure 4:**
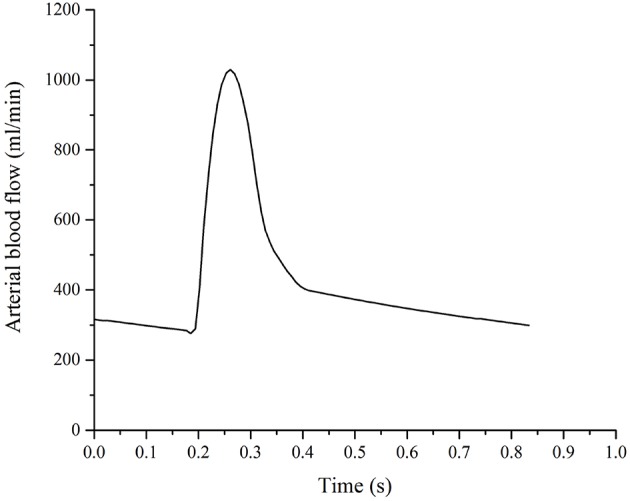
Arterial blood supply to the brain, which is applied as a flux boundary condition of the arterial blood compartment at the cortical surface.

The details of the boundary conditions explained from a physiological perspective can be found in previous publications (Tully and Ventikos, 2011; Vardakis et al., 2013; Guo et al., 2018); a summary is given here and their values can be found in Table 2. One of the boundary conditions that is closely related to the modeling in this paper is the arterial blood flow at the cortical surface (Equation 20). The arterial blood supply to the brain is mainly provided by two pairs of arteries—internal carotid arteries and vertebral arteries (Tortora and Derrickson, 2009). Due to the lack of explicit characterization of vasculature in the 4-MPET model, the arterial blood supply to the brain is simplified into a flux boundary condition (Neumann boundary condition) *Q*_*a*_ at the cortical surface, which is applied as pulsatile waveforms (Figure 4). The numerical simulations run 50 cycles of arterial blood waveforms to reach a periodic steady state; only the output data from the final steady state are used for validation in section Results and Discussion.

**Table 2:** Poroelastic parameters used in the 4-MPET modeling.

**Parameters**	**Values**	**Units**	**Parameters**	**Values**	**Units**
*α_*a*_*_**c**_	0.25		*ω_*ac*_*	1.5 × 10^−7^	m^2^N^−1^s^−1^
*α_*e*_*	0.49		*ω_*cv*_*	1.5 × 10^−7^	m^2^N^−1^s^−1^
*α_*v*_*	0.01		*ω_*ev*_*	1.0 × 10^−6^	m^2^N^−1^s^−1^
*λ_*g*_*	505	Pa	*ω_*ce*_*	1.0 × 10^−8^	m^2^N^−1^s^−1^
*G_*g*_*	216	Pa	*p_*bp*_*	650	Pa
*λ_*w*_*	1,010	Pa	*Q_*p*_*	5.8 × 10^−9^	m^3^s^−1^
*G_*w*_*	432	Pa	*Q*_0_	5.8 × 10^−9^	m^3^s^−1^
*S_*a*_*_**c**_	2.9 × 10^−4^	m^2^N^−1^	*κ_*c*→*vent*_*	6.0 × 10^−4^	m^6^N^−1^s^−1^
*S_*e*_*	3.9 × 10^−4^	m^2^N^−1^	*μ_*e*_*	8.9 × 10^−4^	m^−2^Ns
*S_*v*_*	1.5 × 10^−5^	m^2^N^−1^	*R*	8.5 × 10^13^	m^−3^
*k_*a*_*,_**e*, *v**_	1.0 × 10^−10^	m^2^	*d*	4.0 × 10^−3^	m
*k_*cg*_*	1.0 × 10^−8^	m^2^	*L*	7.0 × 10^−2^	m
*k_*cw*_*	1.0 × 10^−10^	m^2^			

For the arteriole/capillary blood compartment, the production of CSF from the blood results in a pressure drop in the arteriole/capillary blood (Equation 23), where κ_*c*→*vent*_ is the resistance of the flow from the capillary network to the ventricles (through the choroid plexus), and *Q*_*p*_ is the rate of CSF production. Two assumptions are adopted in this boundary condition. First, there is no separation of the two extracellular fluid compartments in the brain—the cerebrospinal fluid (CSF) and the interstitial fluid (ISF) in the 4-MPET model, which assumes that all of the CSF/ISF is produced within the ventricles from blood at a production rate *Q*_*p*_. However, it has been reported that ~20% of CSF in the human brain originates from brain ISF (Edsbagge et al., 2004; Lei et al., 2017). In the current 4-MPET model, this part of CSF production is implicitly embedded in the combined CSF/ISF compartment. Second, the main site of CSF production in the ventricles is the choroid plexus, which is a highly vascularized tissue located within each ventricle of the brain and develops from several locations along the dorsal axis of the neural tube (Lun et al., 2015). The classical hypothesis involves the production of CSF at the choroid plexus of the lateral, third, and fourth ventricles. However, it is still speculative as to the exact proportions of CSF production in the various choroid plexus sites (Gupta et al., 2009; Vardakis et al., 2013). The 4-MPET model simplifies the production of CSF as a uniform distribution on the entire ventricular wall, instead of at specific locations. This simplification is consistent with the homogenization approach adopted for the 4-MPET model.

The CSF/ISF compartment has a Dirichlet boundary condition at the cortical surface and a mixed boundary condition at the ventricular wall. At the cortical surface, the boundary condition (Equation 24) represents the pressure rise resulted from the absorption of CSF into the venous network, where *p*_*bp*_ is the venous blood pressure at the cortical surface, μ_*e*_ is the viscosity of CSF, *R* is the resistance to outflow through the arachnoid granulations, and *Q*_0_ is the out-flux of CSF at the skull (the rate of absorption *Q*_0_ is assumed to be equal to the production rate *Q*_*p*_ in the quasi-steady approach). At the ventricular wall, the boundary condition (Equation 25) represents the conservation of the mass of fluid in the ventricles. Within the ventricles, it is assumed that any CSF that is produced (*Q*_*p*_) and does not flow through the cerebral aqueduct (Poiseuille's law) or the parenchyma must accumulate within the ventricles, where *d* and *L* are the diameter and length of the cerebral aqueduct, respectively, *r*_1_ is the distance from the center to the ventricular wall, and $u_{1}^{n}$ is the displacement at the ventricular wall.

Two subject-specific brain models are simulated in this paper—one healthy control and one patient with unilateral (right) middle cerebral artery (MCA) stenosis. Unilateral MCA stenosis and other intracranial artery stenosis are common causes of ischemic stroke (Mazighi et al., 2006). Previous research reported a reduced lumen diameter of <50% between normal and MCA stenosis by Transcranial Doppler (TCD) (Wang et al., 2014). To account for the reduced blood supply to the right cerebrum, the arterial blood boundary condition at the cortical surface is decreased to 50% for the patient.

Table 2 gives the poroelastic parameters used in the numerical simulations of this paper. These parameters are introduced in the traditional consolidation theory of poroelastic media (Biot, 1941; Wilson and Aifantis, 1982), and also interpreted from a physiological sense for the cerebral environment. Most of the parameters have been used before and the detailed descriptions can be found in previous studies (Tully and Ventikos, 2011; Vardakis et al., 2013; Guo et al., 2018).

The main difference of parameters compared with previous research of MPET modeling (Tully and Ventikos, 2011; Vardakis et al., 2013; Guo et al., 2018) is the differentiation between the gray matter and the white matter. In previous work, the entire brain parenchyma was treated as a homogeneous domain from a mechanical perspective; therefore, there was only one value for elastic constants—shear modulus *G* and Lamé's constant λ (Table 2), respectively. An assumption of single permeability (*k*_*c*_) was also adopted for the arteriole/capillary fluid network. In this paper, the segmentation of T1w MR images (section T1-Weighted MRI) defines separate regions for the gray matter and the white matter (Figure 3); therefore, different values are assigned for the mechanical properties of the gray matter and the white matter. More specifically, previous work of MPET modeling used a Young's modulus of 584 Pa (Taylor and Miller, 2004) for the entire brain. However, experiments have found the gray matter is significantly more compliant than the white matter (Finan et al., 2017; Testu et al., 2017). Therefore, in this paper the Young's modulus of the white matter is twice the value for the gray matter (Weickenmeier et al., 2017), which results in higher values of shear modulus *G* and Lamé's constant λ for the white matter (with subscript *w*) than the gray matter (with subscript *g*).

Another difference from previous MPET simulations is that different values of the permeability of the arteriole/capillary fluid network (*k*_*c*_) are assigned for the white matter and the gray matter. In the theory of poroelasticity, the permeability defines the ability of the porous medium to transmit fluids (Wang, 2000). In general, higher permeability enables the fluid to flow faster through the porous medium according to the Darcy's law (Equation 18). The focus of this paper is to validate the blood diffusion (Darcy velocity of the arteriole/capillary compartment) by CBF data from ASL images, so it is important to characterize the permeability associated with the arteriole/capillary compartment at a more detailed level than the other three fluid compartments. The normal average cerebral blood flow (CBF) in adult humans is about 50 ml/100 g/min (Lassen, 1985; Fantini et al., 2016) with lower values in the white matter and higher values in the gray matter (Vavilala et al., 2002); therefore, in this paper the permeability of the arteriole/capillary compartment (*k*_*c*_) in the gray matter (with subscript g) is set to be 100 times the value for the white matter (with subscript *w*).

## Results and Discussion

The cerebral blood flow (CBF) data from arterial spin labeling (ASL) images and the numerical results obtained from 4-MPET modeling are compared in this section. The 4-MPET model used for numerical simulations can output a wide range of results. The focus of this paper is to validate the blood perfusion; therefore, only the Darcy velocity (filtration velocity) of the arteriole/capillary compartment is shown in this section.

The values of CBF data and blood perfusion from 4-MPET modeling cannot be compared directly (Guibert et al., 2013). The unit of CBF normally is ml/100 g/min, which means the blood volume that flows per unit mass per unit time in brain tissue (Fantini et al., 2016), whereas the unit of Darcy velocity (filtration velocity) is m/s, which means the volume of blood crossing a unit area per unit time. The unit of the filtration velocity (m/s) can be converted to the unit of CBF (ml/100 g/min) by dividing it by the density of the brain tissue 1.0 g/cm^3^ (Barber et al., 1970) and a reasonable length scale at the order of the size of a gyrus (1 cm) (Im et al., 2008).

**Figure 5:**
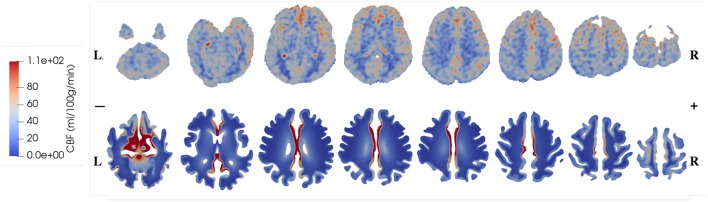
Comparison of CBF data obtained from ASL images (upper row) and blood perfusion from 4-MPET modeling (lower row) for the healthy control.

**Figure 6:**
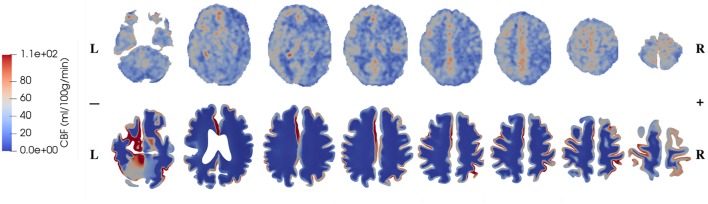
Comparison of CBF data obtained from ASL images (upper row) and blood perfusion from 4-MPET modeling (lower row) for the patient with unilateral (right) MCA stenosis.

The CBF data and blood perfusion results from 4-MPET modeling for the healthy control and the patient with unilateral (right) middle cerebral artery (MCA) stenosis are shown in Figures 5, 6, respectively. The red color represents regions of high blood perfusion and the blue color represents low blood perfusion. There are several similarities that can be seen from the comparison. The first one is that blood perfusion is higher in the gray matter than in the white matter, which means a higher permeability value of the arteriole/capillary compartment in the gray matter is necessary in order to capture this difference. Numerical simulations using identical permeability for the gray matter and the white matter (results are not shown here) demonstrate that different blood perfusion magnitudes in the gray matter and the white matter cannot be reflected in these simulations. In Figures 5, 6, the maximum value in the 4-MPET modeling results is about 2.0 × 10^−4^ m/s, which is equivalent to 1.2 × 10^2^ ml/100 g/min—within the same order of magnitude of the maximum value on the CBF maps, 1.1 × 10^2^ ml/100 g/min.

In numerical modeling, the blood perfusion is taken as the Darcy velocity of the arteriole/capillary compartment; therefore for a more quantitative validation it can also be compared with published data of the blood flow velocity in capillaries of the brain. For example, in a review article, Hudetz (1997) suggested that the red blood cell (RBC) velocity falls in the range of 5 × 10^−4^-1.8 × 10^−3^ m/s within the cerebral capillary network; Hadjistassou et al. (2015) reported a mean capillary blood velocity of 7.3 × 10^−4^ m/s (Lücker et al., 2018); used the red blood cell velocity between 4 × 10^−4^ and 2 × 10^−3^ m/s in their computation model. It can be seen from Figures 5, 6 that the blood perfusion results obtained from 4-MPET modeling are at the same order of magnitude with these published data.

**Figure 7:**
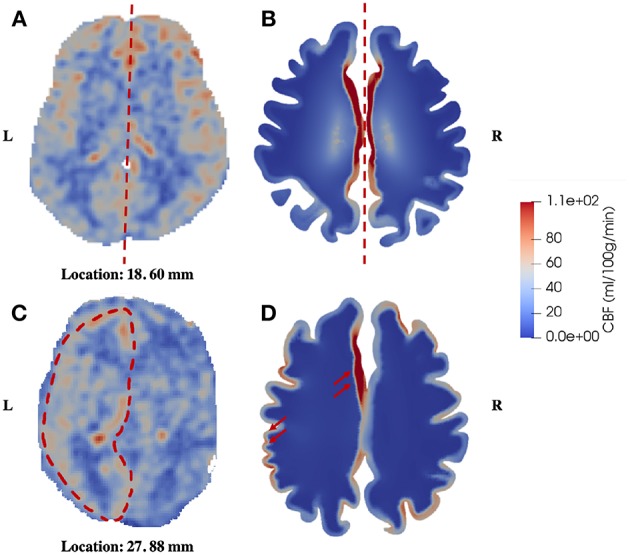
Comparison of symmetric and asymmetric blood perfusion between the healthy control and the patient with unilateral MCA stenosis. **(A)** CBF—healthy control; **(B)** Blood perfusion—healthy control; **(C)** CBF—patient; **(D)** Blood perfusion—patient, the red arrows point to high blood perfusion in the gray matter.

The second similarity that can be seen from the comparison is that there is clear symmetry between left and right cerebrums in the healthy subject (Figure 5), whereas the patient with unilateral (right) MCA stenosis shows lower blood perfusion in the right cerebrum (Figure 6). This is consistent with previous findings (Liu and Li, 2016; Lyu et al., 2016; Lou et al., 2019), which reported that patients with unilateral MCA stenosis have significantly lower CBF in the hemispheres of the stenotic side. A more detailed comparison is shown in Figure 7, where one slice in the horizontal plane is taken from the healthy control and the patient, respectively. The comparison shows that the healthy control has symmetric distribution of blood perfusion; however, the patient with unilateral (right) MCA stenosis has normal blood perfusion in the left cerebrum (highlighted by red dashed lines and arrows) but lower perfusion in the stenotic side (right). This also demonstrates that the reduced arterial blood flow boundary condition applied on the right cortical surface (section Boundary Conditions and Poroelastic Parameters) is correctly reflected in the output of blood perfusion, which means that the coupling directional flow between the arterial blood compartment and the arteriole/capillary blood compartment (Figure 1) is well-defined, and is able to capture different flows.

**Figure 8:**
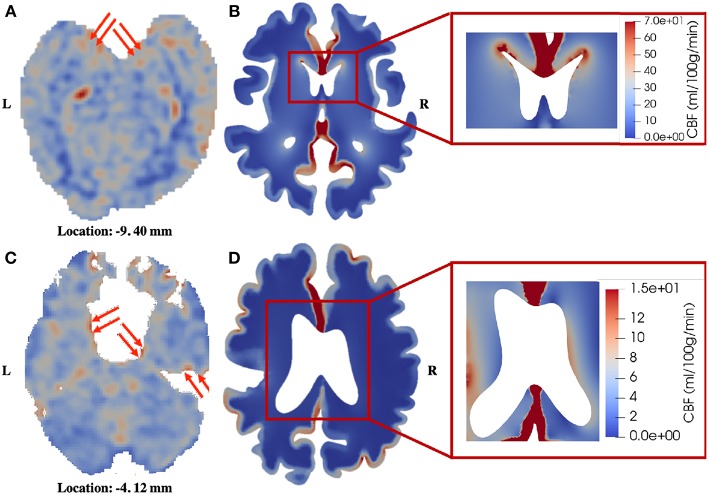
Comparison of blood perfusion in the periventricular region. **(A)** CBF—healthy control; **(B)** Blood perfusion—healthy control; **(C)** CBF—patient; **(D)** Blood perfusion—patient. The red arrows point to high blood perfusion. It should be noted that in order to highlight the local variances in the periventricular region, the color bar in the insets is different from the one used for the entire brain slice.

The third similarity is that the periventricular region shows relatively higher perfusion in the patient with unilateral MCA stenosis, which can also be identified in the healthy control (Figure 8). In 4-MPET modeling, this feature is partly contributed by the local high magnitude of tissue strain in the periventricular region, which demonstrates that the coupling between solid deformation and fluid flow plays an important role in capturing the correct mechanical response. The local variances of blood perfusion in the periventricular region from 4-MPET modeling are shown in the insets of Figures 8B,D. It should be noted that the ventricles are not completely visible on the CBF maps due to resolution characteristics; however, local regions of high perfusion can still be identified around the visible parts of the ventricular wall, which are highlighted by red arrows.

It is worth pointing out that very low perfusion can be seen in the white matter of 4-MPET modeling results, which is mainly due to the separation of the arterial blood and the arteriole/capillary blood compartments, and the assumption of homogenization used in the 4-MPET modeling. The ASL technique uses arterial blood water as an endogenous tracer to measure CBF, whereas in the 4-MPET model the arterial blood is further segmented into a high-pressure arterial network and a lower-pressure arteriole/capillary network (Tully and Ventikos, 2011) at two separate scales. Therefore, the relatively higher velocity of arterial blood cannot be seen in the arteriole/capillary network. The other reason is the assumption of homogenization adopted in the 4-MPET model, which means that there is no explicit characterization of the vasculature in the simulations so the regions of high perfusion are smoothed out. Hence, the very low perfusion in the white matter does not correspond to very low ASL signal that would be incompatible with a live person.

The numerical results presented in this paper mainly show qualitative validation, with several limitations that need to be addressed. First, it can be seen from Figures 7, 8 that the CBF data from ASL images exhibit higher degree of heterogeneity in the parenchyma than the blood perfusion results from 4-MPET modeling. The main reason for this is that there is no explicit characterization of subcortical structures and vasculature as input conditions for numerical modeling; therefore, the heterogeneous distribution of blood perfusion is smoothed out due to this assumption of homogenization. Another reason is that some of the high-perfusion regions found on the CBF maps are large arteries, not arterioles; therefore the blood velocity is considerably higher than the surroundings. One possible solution is to assign different values of Young's modulus by simply allowing for some heterogeneity within a small range in the parenchyma, in addition to differentiating between the white and gray matter. Another possible solution to improve this is to use heterogeneous properties (e.g., shear modulus) obtained from magnetic resonance elastography (MRE), which is a non-invasive imaging method to quantitatively assess the mechanical properties of biological tissue *in vivo* (Green et al., 2008). It is also worth noting that spatially varying permeability tensors are not incorporated for the CSF/ISF compartment in the current study, which is another reason for the lack of heterogeneity in the numerical results. Second, only two subjects are included in the validation, which makes the sample too small to conduct a thorough statistical analysis. Once more data are collected, machine learning, such as the differential evolution (DE) algorithm for non-linear optimization of finite element solutions (Storn and Price, 1997; Cao et al., 2006), can be used to optimize the poroelastic parameters used in 4-MPET modeling. Third, the partial validation of the MPET model in this paper only focuses on the arteriole/capillary compartment, which is not necessarily the most comprehensive scenario to demonstrate the advantages of the MPET model as a whole—ideally, experimental, or clinical data should be collected to show the coupling effects between fluid compartments. Unfortunately, such data are not available at the moment. Therefore, the strategy is to validate the fluid compartments in the MPET model one by one, and then validate the coupling effects once the required data becomes available.

## Conclusions

The paper demonstrates the extent to which the four-network poroelastic model (4-MPET) agrees with arterial spin labeling (ASL) images in terms of blood perfusion. Several similarities can be found between 4-MPET modeling and cerebral blood flow (CBF) data obtained from ASL images. First, the blood perfusion is higher in the gray matter than in the white matter for both the healthy control and the patient with unilateral middle cerebral artery (MCA) stenosis. Second, the healthy control shows symmetric distribution of blood perfusion, whereas the patient has lower perfusion in the stenotic side of the brain. Third, the blood perfusion is relatively higher in the local periventricular region for both the healthy control and the patient with unilateral MCA stenosis. Although the partial validation is presented mainly in a qualitative way, it is one important step in a series of tests toward the full validation of the 4-MPET model. This paper also explains the need for more experimental and clinical data to optimize the boundary conditions and parameters used in numerical modeling. The potential exists to use the 4-MPET modeling workflow as a testing bed for hypotheses and new theories in neuroscience research.

## Ethics Statement

This study was carried out in accordance with the recommendations of the institutional and/or national research committee with written informed consent from all subjects. All subjects gave written informed consent in accordance with the Declaration of Helsinki. The protocol was approved by the ethics committee of the PLA General Hospital.

## Author Contributions

LG, ZL, JL, YM, JV, DC, CH, XL, and YV conceived of the study, designed the research, and coordinated the writing of the manuscript. YV was the senior author of this paper and coordinated the entire research. LG and ZL contributed in the development of the workflow for numerical simulations. JL collected and processed the clinical data. YM and JV helped the discussion and revision of the manuscript. All authors gave final approval for publication.

### Conflict of Interest Statement

The authors declare that the research was conducted in the absence of any commercial or financial relationships that could be construed as a potential conflict of interest. The handling editor is currently organizing a Research Topic with one of the authors YV, and confirms the absence of any other collaboration.
